# Nutraceutical Potential of Oilseeds and By‐Products (Cakes) of Three Underutilized Malvaceae Trees Grown in Sudan

**DOI:** 10.1002/fsn3.70080

**Published:** 2025-03-05

**Authors:** Munna Tahir Abdel Rahman, Sakina Yagi, Gökhan Zengin, Ozan Emre Eyupoglu, Rosella Spina, Jérémy Grosjean, Ashraf M. A. Abdalla, Dominique Laurain‐Mattar

**Affiliations:** ^1^ Department of Botany, Faculty of Science University of Khartoum Khartoum Sudan; ^2^ Faculty of Clinical Nutrition Sudan International University Khartoum Sudan; ^3^ Department of Biology, Science Faculty Selcuk University Konya Turkey; ^4^ Department of Biochemistry, School of Pharmacy Istanbul Medipol University Turkey; ^5^ INRAE, LAE Université de Lorraine Nancy France; ^6^ Department of Forest Products and Industries, Faculty of Forestry University of Khartoum Khartoum Sudan

**Keywords:** *Adansonia digita*, antioxidant, chemical profile, enzyme inhibitory, *Grewia tenax*, *Thespesia garckeana*

## Abstract

The nutraceutical potential of seed oils and cakes of *Adansonia digitata* , *Grewia tenax*, and *Thespesia garckeana* was evaluated by determining their chemical profile and examining their antioxidant and enzyme inhibitory properties. Oils of *G. tenax* and *T. garckeana* were rich in polyunsaturated fatty acids. The cake methanolic extract of *T. garckeana* revealed the highest antiradical (44.67 mg trolox equivalent (TE)/g extract) and metal chelating (26.38 mg EDTA equivalent/g extract) properties, while the oil of *G. tenax* displayed the highest Cu^++^ (180.62 mg TE/g extract) and Fe^+++^ (82.07 mg TE/g extract) reducing capacity. Pelargonidin and rutin were the dominant antioxidant compounds. The oil of *A. digitata* displayed the highest anti‐acetylcholinesterase (2.44 mg galantamine equivalent (GALAE)/g extract) and butyrylcholinesterase (2.10 mg GALAE/g extract) activity, while its cake exhibited the best α‐glucosidase inhibitory activity (1.46 acarbose equivalent (ACAE)/g extract). The cake of *T. garckeana* exerted the highest α‐amylase inhibitory effect (0.71 mmol ACAE/g extract). The highest anti‐tyrosinase activity (10.88 mg kojic acid equivalent/g extract) was recorded from the cake of *G. tenax* . These results indicated that these seeds could be a rich source of antioxidants that target diseases associated with oxidative stress, like diabetes and certain neurological disorders.

## Graphical abstract

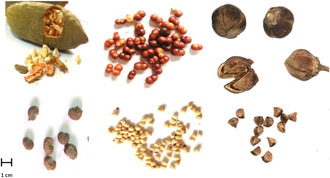

## 1 Introduction

Food insecurity is widespread in many regions of the world, particularly in low‐income communities. Around 26.4% of the world's population suffers from food insecurity and, therefore, malnutrition (FAO 2019). Wild edible plants form an important component of traditional ethnic foods in various regions of the world and are primarily consumed by many communities in rural villages during harsh times of climate‐related environmental and humanitarian crises (Mokria et al. 2022). Several studies have highlighted the important roles of wild edible plants as a source of functional foods with health benefits beyond basic nutrition (Pinela et al. 2016, 2017; Plasek et al. 2019). Food products with health benefits, including the prevention and treatment of diseases, are called nutraceuticals (Damián et al. 2022). Molecules with nutraceutical potential include polyphenols, alkaloids, terpenes, SFAs, vitamins, and trace elements (Damián et al. 2022). Furthermore, plant‐based waste and by‐products were shown to be rich in nutrients and bioactive compounds and could contribute to a more sustainable and efficient food production system (Aït‐Kaddour et al. 2024). For example, lycopene extracted from tomato skins waste by extra virgin olive oil as a green solvent was found to exert significant biological activities and was suggested as a novel food, dietary supplement, and nutraceutical in the human diet (Marinaccio et al. 2024). Grape pomace was found to be rich in polyphenols and possess significant antioxidants and anti‐tyrosinase activities (Marinaccio et al. 2025).

Oilseeds are consumed for their oil, nutritional, and therapeutic potential (Jayaraj et al. 2020). After oil extraction, a by‐product called oil cake or meal is obtained. Oilseed cakes are rich in protein, vitamins, minerals, organic acids, amino acids, surfactants, pigments, and enzymes. Recently, they are considered a good source of natural bioactive substances with potential health benefits (Saeed et al. 2022; Ancuța and Sonia 2020). Additionally, due to their low price and rich nutrient content, many food products and diet formulas for undernourished people are prepared from oilseed cakes (Kotecka‐Majchrzak et al. 2020; Usman et al. 2023).

The demand for wild edible plants for their consumption and medicinal properties has been increasing locally and internationally in Sudan and other countries worldwide (Gebauer et al. 2016; Damián et al. 2022). The family Malvaceae is represented by 243 genera and approximately 4225 species distributed worldwide, particularly in tropical regions (Simpson 2010). The family was previously included in 4 families, namely, Malvaceae, Bombacaceae, Sterculiaceae, and Tiliaceae. However, phylogenetic studies and molecular analysis confirmed their Malvalean identity but indicated that these families are largely nonmonophyletic. So, they merged them into one family, the Malvaceae, and subdivided it into nine subfamilies (Bayer et al. 1999; Bayer and Kubitzki 2003; Baum et al. 2004; Cvetković et al. 2021). In Sudan, there are many plants belonging to the family Malvaceae valued for their economic, food, and medicinal properties. For example, *Adansonia digitata* L. (subfamily Bombacoideae) is an economically important tree with variable uses. The tree is known as Tabaldy and the fruit as Gongolaise. In traditional medicine, the fruit is used to treat diarrhea and dysentery, fever, stomachache, giardiasis, and pain after birth (Yagi 2021). The fruit pulp is edible and proven to possess antioxidant and enzyme inhibition activities, and it is rich in vitamin C and phenolics, particularly catechin and epicatechin (Hussain et al. 2019). Another example is *Thespesia garckeana* F.Hoffm. (syn. *Azanza garckeana* (F.Hoffm.) Exell & Hillc.) (subfamily Malvoideae). The fruit, commonly known as Jakjak, is edible and has a sweet taste and glutinous slime. A porridge is also prepared from the fruit pulp (Suliman et al. 2012). Studies revealed that it has anti‐arthritic, antimicrobial, wound healing, fecundity, and reproductive activities, among others (Yusuf et al. 2020; Bukar et al. 2021; Bukar et al. 2020; Dikko et al. 2016; Lawal et al. 2022; Masila et al. 2015). Phenolic compounds, terpenes, and sterols were isolated from different parts (Masila et al. 2015; Lawal et al. 2022). One more plant is *Grewia tenax* (Forssk.) Fiori (subfamily Grewioideae) and locally known as Godaim. A light porridge is prepared from the fruit for lactating mothers. It is also used to treat anemia, malaria, and as a tonic (Issa et al. 2018). *G. tenax* was found to possess hepatoprotection, antioxidant, antidiarrhoeal, antitumor and herbicidal, and antimicrobial activities, among others (Kumar et al. 2022).

Despite the importance of wild edible plant seeds in many parts of Sudan as a source of food, there is a lack of information about their chemical composition, and no attention has been given to their role as a main source of nutraceuticals. Additionally, as previously stated, and due to their low price and rich nutrient content, oilseed cakes can be an alternative ingredient for many food products and dietary supplements. *A. digitata, G. tenax*, *and T. garckeana* are widely grown in Western Sudan, and as mentioned above, their fruit pulps are commonly consumed by the people in Sudan and in other African countries; however, the seeds are left as waste, and the potential industrial applications of these seeds have not been explored yet. Therefore, research on these seeds and the expansion of their potential application is of great significance, and the current study was designed to evaluate the chemical constituents, antioxidant, and enzyme inhibition properties of their oils and cake extracts.

## 2 Materials and Methods

### 2.1 Plant Materials

**FIGURE 1:**
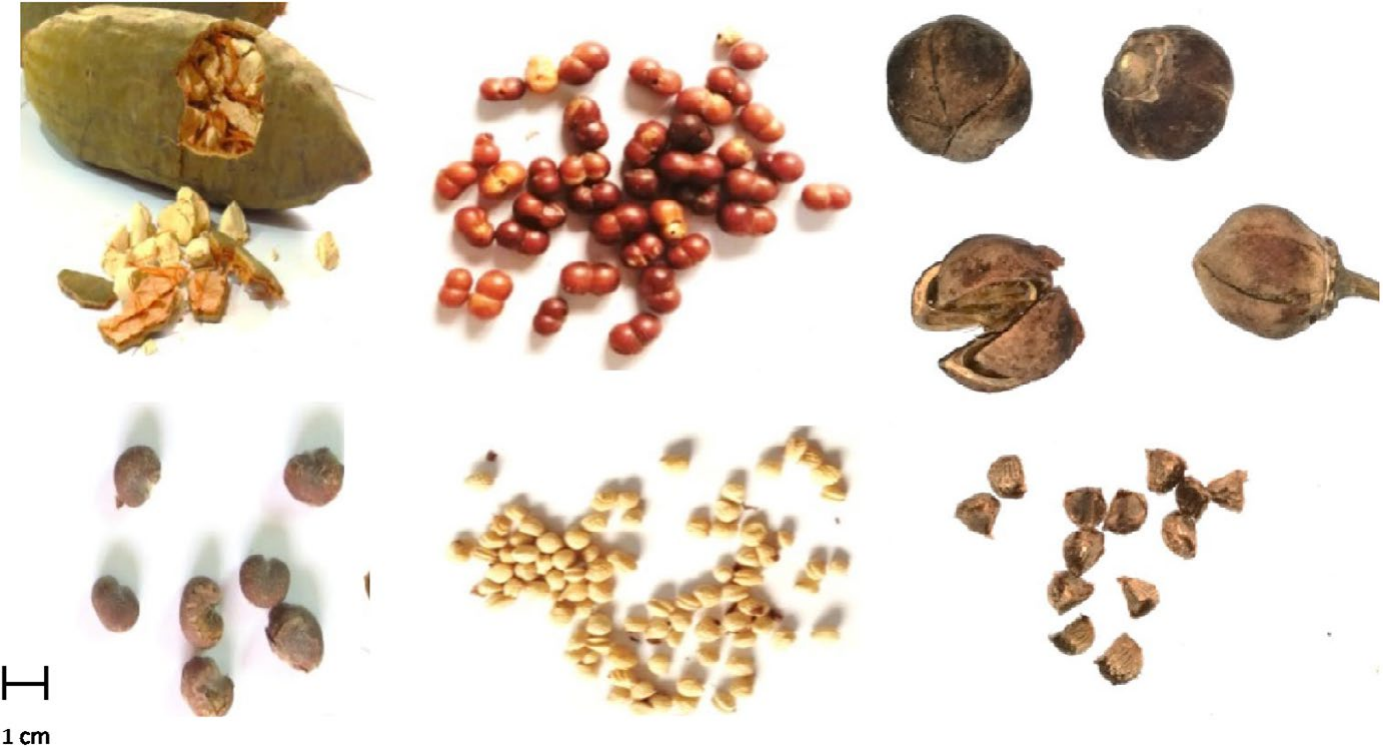
Fruits and seeds of the investigated plants.

Fruits of *A. digitata, G. tenax*, and *T. garckeana* were collected from wild trees growing around El Obeid City, Kordofan State (latitude 13.18421 and longitude 30.21669), Sudan. The fruits of each plant, all free from injuries, were randomly picked on different days and were mixed into a single batch of 500 g. The plants were identified and authenticated at the Department of Botany, Faculty of Science, University of Khartoum. Voucher specimens (AD/619/BH, GT/919/HB and TG/119/HB for *A. digitata, G. tenax*, and *T. garckeana*, respectively) were deposited at the Herbarium of the Botany Department. Healthy dry fruits from each sample were ruptured, and seeds were manually picked up, soaked in ionized water for 30 min., and pulped separated from the seeds by maceration and washing with ionized water. Seeds were air‐dried under shade till a constant mass was obtained and then ground into powder using an electric grinder. The obtained powdered seeds were kept at 20°C until subsequent analyses. Photos of fruit samples and their seeds are presented in Figure 1.

### 2.2 Preparation of Extracts

The fine powder (20 g) of seeds was extracted successively by maceration in n‐hexane (400 mL) (considered as oil extract) and methanol (400 mL) using a shaker apparatus, at room temperature, filtered, and then the solvents were evaporated. The resultant dry extracts from each sample were weighed and stored in the refrigerator at 4°C until their usage.

### 2.3 Fatty Acids Analysis

Fatty acids present in oils were converted to fatty acid methyl esters as described by Carvalho and Malcata (2005). The method involves alkaline hydrolysis with 0.5 M sodium hydroxide at 100°C for 5 min., followed by acidic transmethylation with 12% (v/v) boron trifluoride in methanol at 100°C for 3 min. Phase separation was achieved with a solution of saturated sodium chloride using hexane as an extracting solvent.

#### 2.3.1 GC/MS Analysis

The chemical profile of oil seeds was determined by gas chromatography (GC) (Thermo Scientific Trace 1310) coupled with a mass spectrometry system (MS) (Thermo Scientific TSQ 9000 GC‐MSMS) according to the method described by Hemmati et al. (2020). Detailed methodology is found in the Supporting Information.

### 2.4 Determination of Total Phenolic and Flavonoid Contents in Oils and Cake Methanolic Extracts

#### 2.4.1 Extraction of Polyphenol From Oil

Extraction of polyphenol content in oils (obtained by n‐hexane as described in 2.2) was realized according to the method described by Marfil et al. (2011). Briefly, 1.5 g of the oil sample was dissolved in 2 mL of hexane and then subjected to liquid–liquid extraction with methanol/water (40:60, v/v). The oily extract was filtered, and the filtrate was used for further analysis.

#### 2.4.2 Assay for Total Phenolic and Flavonoid Contents

The total content of phenols and flavonoids in oils and cake methanolic extracts was assessed following the procedures described in the paper (Slinkard and Singleton 1977).

### 2.5 Antioxidant Tests

#### 2.5.1 Antioxidant Tests by Spectrophotometric Methods

Antioxidant assays were conducted using the previously reported method (Grochowski et al. 2017). For the 2,2‐diphenyl‐1‐picrylhydrazyl (DPPH), 2,2′‐azino‐bis (3‐ethylbenzothiazoline‐6‐sulfonic acid) (ABTS) radical scavenging, Cupric reducing antioxidant capacity (CUPRAC), and ferric reducing antioxidant power (FRAP) tests, the results were expressed as mg of Trolox equivalents (TE)/ g extract. In the phosphomolybdenum (PBD) assay, the antioxidant potential was quantified in terms of mmol of TE/g extract. Metal chelating activity (MCA) was expressed as mg of disodium edetate equivalents (EDTAE)/g extract.

#### 2.5.2 On‐Line HPLC‐Antioxidant Methodologies

The antioxidant potential of all the extracts was evaluated using online HPLC‐based assays (FRAP, DPPH, ABTS, and CUPRAC) (Benzie and Strain 1999; Re et al. 1999; Koleva et al. 2000; Apak et al. 2004; Sinan et al. 2021). Four distinct HPLC methodologies—HPLC‐FRAP, HPLC‐DPPH, HPLC‐ABTS, and HPLC‐CUPRAC—were employed separately for antioxidant detection and activity determination. In HPLC‐FRAP, fresh FRAP reagent was used with detection signals at 280 nm for DAD phenolic peaks and 595 nm for UV antioxidant peaks. At the same time, HPLC‐DPPH and HPLC‐ABTS utilized negative post‐column detection with fresh DPPH and ABTS radical reagents, respectively, resulting in UV peak chromatograms at 517 and 734 nm alongside DAD 280 nm peak chromatograms. Additionally, HPLC‐CUPRAC allowed negative post‐column detection and involved the reduction of Cu(II)‐Neocuproine reagent in a redox reaction, with simultaneous UV peak chromatograms at 450 nm and DAD 280 nm peak chromatograms. All analytical parameters are given in the Supporting Information

### 2.6 Enzyme Inhibitory Tests

The enzyme inhibition experiments for the samples were conducted following previously established protocols (Grochowski et al. 2017). Amylase and glucosidase inhibitory activities were expressed as mmol of acarbose equivalents (ACAE)/g extract. Acetylcholinesterase (AChE) and butyrylcholinesterase (BChE) inhibitory activities were expressed as mg of galanthamine equivalents (GALAE)/g extract, while tyrosinase inhibition was expressed as mg of kojic acid equivalents (KAE)/g extract.

### 2.7 Statistical Analysis

Experiments were performed in triplicate, and differences between the extracts were compared using One‐way ANOVA (by Tukey's assay) and GraphPad Prism (version 9.2) was used for the analysis. The *p* value of less than 0.05 was deemed to be statistically significant.

## 3 Results and Discussion

### 3.1 Extractive Yields of Oils and Cake Methanolic Extracts

**FIGURE 2:**
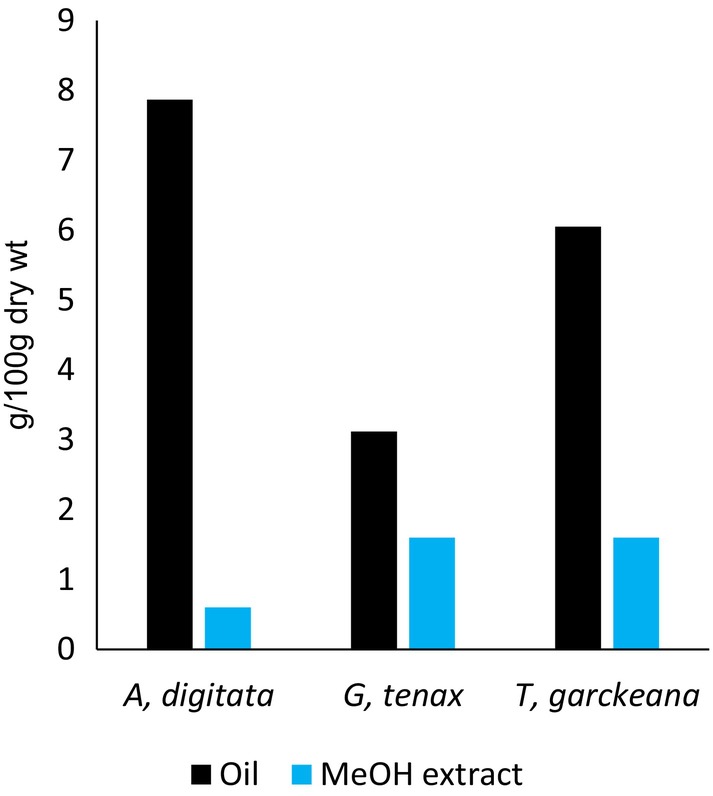
Extractive yield.

Fixed oil was extracted from the seeds of the investigated plants, and then the oilseed cake left was subjected to extraction by maceration in methanol. Yield extracts of fixed oils and cake methanolic extracts are presented in Figure 2. The highest oil yield was obtained from the seed of *A. digitata* (7.87 g/100 g dry wt) followed by that of *T. garckeana* (6.05 g/100 g dry wt) and *G. tenax* (3.12 g/100 g dry wt), respectively. Cake methanolic extracts of *T. garckeana* and *G. tenax* (1.6 g/100 g dry wt) had the same yield, while *A. digitata* displayed the least content (0.6 g/100 g dry wt). Oil yields of the seeds in the present study were lower than those reported for *A. digitata* (12 g/100 g dry wt) (Alnadif et al. 2017) and *G. tenax* (10.7 g/100 g dry wt) (Aboagarib et al. 2016). The yield of oil from *T. garckeana* seeds was higher in the present study than that reported by Suliman et al. (2012) for the whole fruit (1.04 g/100 g dry wt). In fact, yield components and other physicochemical properties of seed oils are highly affected by the genotype and age of the plant, as well as different environmental factors like soil type and climate conditions, among others (Johansson et al. 2000; Rahim et al. 2023). Although the oil yield obtained for these seeds was lower than that of different oilseed crops like soybean (20%) and sunflowers (40%) (Waseem et al. 2017), these seeds could be a new source of oil that deserve further exploration of their nutraceutical potential.

### 3.2 Fatty Acids Profile of the Oils

Chemical profile of the seed oils of *A. digitata, G. tenax*, and *T. garckeana* was mainly composed of saturated fatty acids (SFAs), monounsaturated fatty acids (MUFAs) and polyunsaturated fatty acids (PUFAs). Results are presented in Table 1. The distribution and content of fatty acids varied in the three species. It was noticed that the oils of *G. tenax* and *T. garckeana* were rich in PUFAs (46.50 and 44.71 mg/100 g of oil, respectively) followed by SFAs (32.51 and 33.54 mg/100 g of oil, respectively) but they had a low content of MUFAs (16.49 and 8.59 mg/100 g of oil respectively). The proportions of SFAs, MUFAs, and PUFAs (31.73, 29.54 and 30.75 mg/100 g of oil, respectively) in *A. digitata* oil were relatively close to each other. The profile of fatty acids for the major compounds was similar in the three oils, and they varied for minor components. Palmitic, oleic acid, and linoleic acids were, respectively, the most abundant SFA, MUFA, and PUFA in the three oils. The health benefits of fatty acids were previously reported. For example, a higher level of linoleic acid decreases the systemic level of cholesterol and plays an important role in the prevention of atherosclerosis (den Hartigh 2019). Palmitic acid was shown to possess a selective cytotoxic effect against human leukemia cells and immunomodulatory activity (Boubaker et al. 2018; Song et al. 2023). Arachidic acid regulates type 2 immune responses against intestinal and blood flukes and possesses schistosomicidal and tumoricidal activities (Tallima and El Ridi 2018).

**TABLE 1:** Fatty acids profile (mg/100 g of oil) of the investigated seeds.

Compounds	*A. digitata*	*G. tenax*	*T. garckeana*
SFA			
Pelargonic acid	0.24	—	—
Myristic acid	0.51	0.23	0.58
Palmitic acid	25.87	18.73	25.62
Margaric acid	0.26	0.80	—
Stearic acid	3.05	10.97	3.87
Epoxyoleic acid	0.50	—	—
Arachidic acid	0.83	1.16	3.45
Behenic acid	0.47	—	—
Lignoceric acid	—	0.62	—
Cerotic acid	—	—	0.02
MUFA			
(Z)‐7‐Hexadecenoic acid	0.16	—	—
Palmitoleic acid	—	0.26	—
cis‐10‐Heptadecenoic acid	—	0.12	—
17‐Octadecenoic acid	—	0.07	—
Ricinoleic acid	0.87	1.30	3.89
Trans‐Vaccenic acid	0.39	—	0.09
Oleic acid	27.01	14.23	2.79
cis‐10‐Nonadecenoic acid	1.11	0.21	1.82
Gondoic acid	—	0.30	—
PUFA			
Linoleic acid	29.60	44.21	43.24
γ‐Linoleic acid	—	2.21	—
Linoleic acid cholride	—	0.03	—
Octadecadienoic acid, (Z,Z)	—	0.05	—
7,10‐Octadecadienenoic acid	1.15	—	—
Methyl 6‐cis,9‐cis,11‐trans‐octadecatrienoate	—	—	1.47
⅀ SFA	31.73	32.51	33.54
⅀ MUFA	29.54	16.49	8.59
⅀ PUFA	30.75	46.50	44.71

*Note:* Results expressed as mean of duplicate analysis (*n* = 2).

Abbreviations: MUFA, monounsaturated fatty acids; PUFA, polyunsaturated fatty acids; SFA, saturated fatty acids.

### 3.3 Total Polyphenol (TPC) and Flavonoids (TFC) Contents

The TPC and TFC of the oils and cake methanolic extracts of *A. digitata, G. tenax*, and *T. garckeana* seeds were determined, and results are presented in Table 2. The TPC of the oil was in the range of 0.39–2.59 mg GAE/g, with the highest value recorded for *T. garckeana*. Oils of the other two species had the same TPC. The TPC of the cake methanolic extracts was in the range of 19.28–37.54 mg GAE/g, with the highest and least values obtained, respectively from *T. garckeana* and *A. digitata* . The TFC of oils ranged between 0.14 and 0.29 mg RE/g, with the highest and least values obtained, respectively from *T. garckeana* and *A. digitata* , while that of cake methanolic extract was in the range of 0.95–8.65 mg RE/g, with the highest and least values displayed, respectively by *A. digitata* and *G. tenax*. On comparing these values with previous studies in the literature, it was observed that the TPC and TFC of the methanolic extract of *A. digitata* seed collected from a tree grown in Senegal were comparable for TPC (18.26 mg GAE/g) and higher for TFC (12.82 mg QE/g) than the values obtained in the present study (Ndiaye et al. 2021). For *T. garckeana*, the TPC and TFC were determined for the MeOH extract of fruit pulp, and values (TPC = 260.80–34.32 and TFC = 10.28–13.45 mg/100 g) were far different from those recorded for the seed in the present study, indicating that the seed was richer in phenolics than the fruit pulp (Yusuf et al. 2020; Lawal et al. 2022). Furthermore, this is the first report on the TPC and TFC of *G. tenax* ; however, the values for *G. sapida* methanolic extract of the fruit were higher (TPC = 294.353 mg GAE/g; TFC = 116.95 mg QE/g) than those obtained for *G. tenax* seed in the present study (Islary et al. 2016). Moreover, the presence of phenolic compounds in oilseeds improves their oxidative stability, thus potentially extending their shelf life, besides their beneficial effect on human health (Morya et al. 2022). They also affect their organoleptic, nutraceutical, and functional properties (Zhang et al. 2022). Overall, the higher TPC and TFC in cake extracts than their respective oils could be mainly due to the fact that they were of polar nature and preferentially recovered with methanol (Hina et al. 2016). Consequently, cakes could have a great potential to be used as an inexpensive, valuable source of phenolic compounds suitable for numerous applications.

**TABLE 2:** Total phenolic and flavonoid contents of oil and cake methanolic extracts of the seeds.

Plant species	Extracts	Total phenolic content (mg GAE/g extract)	Total flavonoid content (mg RE/g extract)
*Adansonia digitata*	Oil	0.39 ± 0.12^e^	0.14 ± 1.92^e^
	MeOH	19.28 ± 0.15^c^	8.65 ± 1.45^a^
*Grewia tenax*	Oil	0.39 ± 0.26^e^	0.21 ± 1.45^d^
	MeOH	23.95 ± 0.15^b^	0.95 ± 0.04^c^
*Thespesia garckeana*	Oil	2.59 ± 0.39^d^	0.29 ± 0.75^d^
	MeOH	37.54 ± 0.53^a^	7.67 ± 0.58^b^

*Note:* Different letters in the same column indicate significant differences in the extracts (*p* < 0.05).

Abbreviations: GAE: Gallic acid equivalent; RE: Rutin equivalent.

* Values are reported as mean ± SD of three parallel measurements.

### 3.4 Antioxidant Activity

Methods used to determine the antioxidant properties of herbal extracts are based on hydrogen atom transfer (HAT) reactions, single electron transfer (SET) reactions, in addition to other mechanisms. The SET method included iron (III) ion reducing/antioxidant capacity, copper (II) ion reducing/antioxidant capacity, and the DPPH and ABTS radical scavenging methods. These measurements were performed spectrophotometrically (off‐line) to evaluate the overall activity of mixtures (Prior et al. 2005). Nowadays, the individual bioactive components responsible for the antioxidant activity of complex matrices of plant samples can be determined on‐line by combining the HPLC system with additional pumps and detectors (Arslan Burnaz et al. 2017). In the present study, the antioxidant activity of the oils and cake methanolic extracts was evaluated spectrophotometrically, while the on‐line HPLC‐antioxidant tests were employed to evaluate the activity of the active antioxidants present in the cake methanolic extracts after post‐column reactions.

#### 3.4.1 Off‐Line Antioxidant Activity of Oils and Cake Methanolic Extracts

The antioxidant activity of oils and cake methanolic extracts of *A. digitata, G. tenax*, and *T. garckeana* seeds was evaluated by six complementary assays, and results are presented in Table 3. Oils of *A. digitata* and *G. tenax* did not exert antiradical activity in both the DPPH and ABTS assays, while that of *T. garckeana* showed considerable antiradical activity with a higher capacity to scavenge the TBTS radical (11.22 mg TE/g) than the DPPH one (5.54 mg TE/g). The antiradical activity of *T. garckeana* could be associated with its relatively higher TPC (2.84 mg GAE/g) when compared to that of the other two oils (0.33 mg GAE/g). The DPPH and ABTS radicals scavenging activity of the cake methanolic extracts was in the range of 6.52–44.67 mg TE/g and 16.28–78.54 mg TE/g, respectively. They were in the following order in both assays: *T. garckeana* > *G. tenax* > *A. digitata* , in accordance with their TPC and with previous studies that correlated the antiradical activity of extracts to their TPC.

**TABLE 3:** Antioxidant activity of oil and cake methanolic extracts of seeds.

Plant species	Extracts	DPPH (mg TE/g extract)	ABTS (mg TE/g extract)	CUPRAC (mg TE/g extract)	FRAP (mg TE/g extract)	PBD (mmol TE/g extract)	MCA (mg EDTAE/g extract)
*Adansonia digitata*	Oil	NA	NA	118.69 ± 3.71^b^	56.23 ± 1.14^b^	1.39 ± 0.02^b^	6.81 ± 0.39^c^
	MeOH	6.52 ± 0.36^b^	16.28 ± 0.19^c^	35.72 ± 1.04^d^	16.02 ± 0.24^d^	0.55 ± 0.04^d^	10.32 ± 0.43^b^
*Grewia tenax*	Oil	NA	NA	180.62 ± 2.73^a^	82.07 ± 2.32^a^	NA	1.96 ± 0.01^d^
	MeOH	6.80 ± 0.69^b^	42.37 ± 0.07^b^	39.36 ± 1.03^d^	22.40 ± 0.71^c^	NA	0.56 ± 0.01^e^
*Thespesia garckeana*	Oil	5.54 ± 0.29^c^	11.22 ± 0.42^d^	68.53 ± 1.13^c^	17.02 ± 0.04^d^	1.17 ± 0.00^c^	NA
	MeOH	44.67 ± 0.10^a^	78.54 ± 0.05^a^	174.27 ± 4.57^a^	54.23 ± 0.73^b^	1.80 ± 0.03^a^	26.38 ± 0.50^a^

*Note:* Different letters in the same column indicate significant differences in the extracts (*p* < 0.05).

Abbreviations: ABTS, 2,2′‐azino‐bis(3‐ethylbenzothiazoline‐6‐sulfonic acid); CUPRAC, cupric ion reducing antioxidant capacity; DPPH, 2,2‐diphenyl‐1‐picrylhydrazyl; EDTAE, EDTA equivalent; FRAP, ferric reducing antioxidant power; MCA, metal chelating activity; na, not activePBD, phosphomolybdenum; TE, Trolox equivalent.

* Values are reported as mean ± SD of three parallel measurements.

On the other hand, oils of *G. tenax* and *A. digitata* exerted higher Cu^++^ (4.9‐ and 3.2‐fold, respectively) and Fe^+++^ (3.7 and 3.5‐fold, respectively) reducing power than their respective cake methanolic extracts. On the other hand, the Cu^++^ and Fe^+++^ reducing power of the cake methanolic extract of *T. garckeana* exceeded the oil by 2.5‐ and 3.2‐fold, respectively. All extracts were more effective in reducing Cu^++^ ions than Fe^+++^ ones. The high capacity of *G. tenax* and *A. digitata* oils to reduce ions suggested that other non‐phenolic molecules, including tocopherols, carotenoids, and some minerals (Se and Zn) may also be involved in the observed reducing activity (Gutiérrez‐del‐Río et al. 2021). A previous study on the antioxidant activity of oils from 7 new sunflower lines showed a similar trend; oils did not display antiradical activity but exerted considerable ion reducing capacity (Abdalla et al. 2021).

Furthermore, the cake methanolic extract of *T. garckeana* exhibited the highest metal chelating capacity (26.38 mg EDTAE/g) followed, respectively, by cake methanolic extract (10.32 mg EDTAE/g) and oil (6.81 mg EDTAE/g) of *A. digitata* , while *G. tenax* revealed the least activity (1.96 and 0.56 mg EDTAE/g). Again, the methanolic extract of *T. garckeana* displayed the highest total antioxidant activity (1.80 mmol TE/g) determined by the phosphomolybdenum assay, while both extracts of *G. tenax* were not active. A previous study on *A. digitata* showed that the methanolic extract of the seed possessed considerable antioxidant activity from the DPPH (IC_50_ = 20.19 μg/mL) and FRAP (EC_50_ > 100 μg/mL) assays (Ndiaye et al. 2021). For *T. garckeana*, it was mainly performed for the fruit pulp, where the methanolic and ethyl acetate extracts of *T. garckeana* fruit pulp exerted significant DPPH, ABTS, and hydroxyl scavenging and iron reducing properties (Yusuf et al. 2020; Lawal et al. 2022). The antioxidant activity of *G. tenax* was carried out on the aqueous extract of the root, where it was found to show high anti‐DPPH, anti‐ABTS, and anti‐nitric oxide activities, as well as iron‐reducing properties (Sharma et al. 2016).

#### 3.4.2 On‐Line HPLC‐Antioxidants From the Cake Methanolic Extracts

**FIGURE 3:**
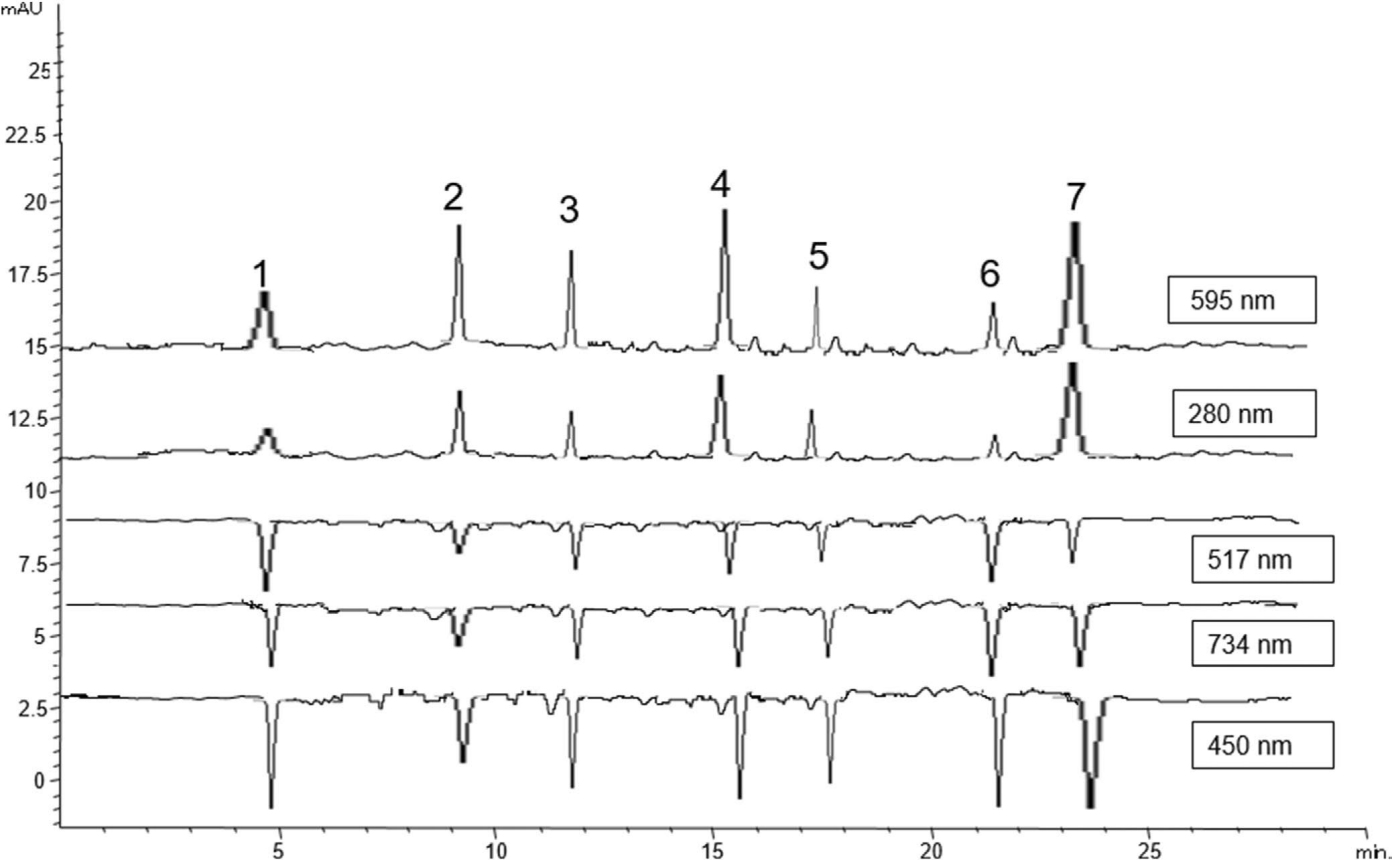
Chromatogram Profile of Defined Peaks at 280 nm, 595 nm, 517 nm, 734 nm, 450 nm (1‐Rutin, 2‐Apigenin, 3‐Quercetin, 4‐Pelargonidin, 5‐Kaempferol, 6‐Epigallocatechine gallate, 7‐Ferulic acid).

The on‐line HPLC‐antioxidant procedure allowed the isolation of the seven selected standards phenolics with satisfactory resolution. To record maximum absorption of the phenolic content, the DAD signal was set to 280 nm, and the UV online‐HPLC–FRAP assay was set at 595 nm to identify the Fe^2+^‐TPTZ complex. The UV online HPLC‐DPPH assay targeted the signal at 517 nm, while that for HPLC‐ABTS and ‐CUPRAC were set at 734 and 450 nm, respectively. This online‐HPLC‐antioxidants system permits real‐time measurement of antioxidants in a sample based on their absorption patterns at specific wavelengths. Data of the four post‐column antioxidant assays of standard mixtures are presented in Table 4 and Table (S1). The seven antioxidants, namely, rutin, apigenin, quercetin, pelargonidin, kaempferol, epigallocatechin gallate, and ferulic acid, were detected within the extracts of the three investigated Malvaceae species at 280, 595, 517, 734, and 450 nm (Figure 3). It was observed that the HPLC–FRAP assay showed higher concentrations of antioxidants compared to the other 3 systems. Pelargonidin was the dominant compound, with the highest concentration recorded for *A. digitata* (85.0 ppm) followed by *T. garckeana* (83.0 ppm) and *G. tenax* (80.0 ppm), respectively, indicating a strong positive response to the FRAP reagent. Ferulic acid was the second major compound in a concentration range of 71.7–78.7 ppm, with the highest and least values obtained from *A. digitata* and *G. tenax* , respectively. Rutin was the principal compound in the other three systems, with a concentration range of 15.5–19.5 ppm in the HPLC‐DPPH assay, 16.5–20.5 ppm in the HPLC‐ABTS assay, and 18.2–23.2 ppm in the HPLC‐CUPRAC assay. Epigallocatechin gallate was the second predominant compound in the three assays (10.5–18.2 ppm), with the highest and least values obtained from *A. digitata* and *G. tenax* , respectively.

**TABLE 4:** Detection and concentration of the compounds defined at 280 nm, 595 nm, 517 nm, 734 nm, and 450 nm for cake methanolic extracts of seeds.

Peak No.	Component names	RT (min.)	Concentrations (ppm)				
			280 nm	595 nm	517 nm	734 nm	450 nm
*Adansonia digitata*							
1	Rutin	5.2	15 ± 0.03^e^	21.3 ± 0.04^b^	19.5 ± 0.02^d^	20.5 ± 0.03^c^	23.2 ± 0.04^a^
2	Apigenin	10.4	57.1 ± 0.04^b^	62.1 ± 0.04^a^	7.5 ± 0.02^d^	8.1 ± 0.03^d^	9 ± 0.04^c^
3	Quercetin	12.3	38.2 ± 0.04^b^	41.7 ± 0.04^a^	9.5 ± 0.02	11.2 ± 0.03	14.2 ± 0.04^c^
4	Pelargonidin	16.5	82.5 ± 0.03^b^	85 ± 0.04^a^	4.2 ± 0.02^e^	6.9 ± 0.03^d^	9 ± 0.04^c^
5	Kaempferol	18.3	40.1 ± 0.04^b^	43.1 ± 0.04^a^	12.8 ± 0.02^d^	13.1 ± 0.03^d^	17 ± 0.04^c^
6	Epigallocatechine gallate	22.2	28.2 ± 0.04^a^	29.8 ± 0.04^a^	14.5 ± 0.02^c^	16.2 ± 0.03^c^	18.2 ± 0.04^b^
7	Ferulic acid	24.5	67.5 ± 0.04^b^	78.7 ± 0.04^a^	5.1 ± 0.02^e^	7.0 ± 0.03^d^	8.2 ± 0.04^c^
*Thespesia garckeana*							
1	Rutin	5.2	14 ± 0.03^c^	20.3 ± 0.04^b^	18.5 ± 0.02^b^	19.5 ± 0.03^ab^	21.2 ± 0.04^a^
2	Apigenin	10.4	55.1 ± 0.04^b^	60.1 ± 0.04^a^	6.5 ± 0.02^e^	7.1 ± 0.03^d^	8 ± 0.04^c^
3	Quercetin	12.3	36.2 ± 0.04^b^	40.7 ± 0.04^a^	8.5 ± 0.02^e^	10.2 ± 0.03^d^	13.2 ± 0.04^c^
4	Pelargonidin	16.5	80.5 ± 0.03^b^	83 ± 0.04^a^	3.2 ± 0.02^e^	5.9 ± 0.03^d^	8 ± 0.04^c^
5	Kaempferol	18.3	39.1 ± 0.04^b^	41.1 ± 0.04^a^	11.8 ± 0.02^d^	12.1 ± 0.03^d^	16 ± 0.04^c^
6	Epigallocatechine gallate	22.2	25.2 ± 0.04^b^	27.8 ± 0.04^a^	13.5 ± 0.02^e^	15.2 ± 0.03^d^	17.2 ± 0.04^c^
7	Ferulic acid	24.5	65.5 ± 0.04^b^	74.7 ± 0.04^a^	4.1 ± 0.02^e^	6.0 ± 0.03^d^	7.2 ± 0.04^c^
*Grewia tenax*							
1	Rutin	5.2	11 ± 0.03^d^	17.3 ± 0.04^b^	15.5 ± 0.02^c^	16.5 ± 0.03^b^	18.2 ± 0.04^a^
2	Apigenin	10.4	52.1 ± 0.04^b^	57.1 ± 0.04^a^	3.5 ± 0.02^d^	4.1 ± 0.03^d^	5 ± 0.04^c^
3	Quercetin	12.3	33.2 ± 0.04^b^	37.7 ± 0.04^a^	5.5 ± 0.02^e^	7.2 ± 0.03^d^	10.2 ± 0.04^c^
4	Pelargonidin	16.5	77.5 ± 0.03^b^	80 ± 0.04^a^	0.25 ± 0.02^e^	2.9 ± 0.03^d^	5 ± 0.04^c^
5	Kaempferol	18.3	36.1 ± 0.04^d^	38.1 ± 0.04^a^	8.8 ± 0.02^d^	9.1 ± 0.03^d^	13 ± 0.04^c^
6	Epigallocatechine gallate	22.2	22.2 ± 0.04^b^	24.8 ± 0.04^a^	10.5 ± 0.02^e^	12.2 ± 0.03^d^	14.2 ± 0.04^c^
7	Ferulic acid	24.5	62.5 ± 0.04^b^	71.7 ± 0.04^a^	1.1 ± 0.02^e^	3.0 ± 0.03^d^	4.2 ± 0.04^c^

*Note:* Different letters in the same column indicate significant differences in the extracts, 95% confidence interval, critical ratio: (*p* < 0.05); ppm, parts per million.

Abbreviations: ±SD, average standard deviation; RT, retention time.

The capacity of pelargonidin to reduce the Fe^+++^ to Fe^++^ in the FRAP assay was suggested to occur through two mechanisms. In the first, pelargonidin offers electrons to Fe^3+^‐TPTZ complexes and thus produces Fe^++^ and flavylium (Moon and Shibamoto 2009). In the second, Fe^+++^ is chelated by two molecules of pelargonidin in the O‐H in the C‐7 position to form complexes (de Souza and De Giovani 2004). Moreover, it was observed that the substituent groups on electron transfer reactions and proton donation capabilities affect significantly the antioxidant reactions of anthocyanins (Sun et al. 2020). Rutin was reported as an antioxidant marker in HPLC‐ABTS (Yang et al. 2023).

UV 280 nm wavelength shows less interference for the detection of many phenolic acids compared to 220 nm and is a wavelength at which many phenolic acids exhibit maximum absorption. However, wavelengths such as 595 nm, 517 nm, 734 nm, and 450 nm can also completely eliminate these interferences through visible region detections after antioxidant reactions and are the maximum wavelength detection regions for antioxidant phenolic acids. Particularly, the FRAP test at 595 nm is valuable for providing positive detection compared to other methods that give negative absorption, is less affected by peak noise, and is effective in measuring both hydrophilic and lipophilic antioxidant activity across a wide spectrum independent of polarity by forming a chelate with iron metal. Indeed, from the HPLC analyses in combination with antioxidant assays, pelargonidin appeared as the major antioxidant showing the highest concentration range (80.0–85.0 ppm) at wavelength 595 nm.

### 3.5 Enzyme Inhibition Activity

Oils and methanolic extracts of *A. digitata, G. tenax*, and *T. garckeana* seeds were evaluated for their enzyme inhibition property against acetylcholinesterase (AChE), butyrylcholinesterase (BChE), tyrosinase (Tyr), α‐amylase, and α‐glucosidase enzymes (Table 5). Oils of the three investigated plants, except that of *T. garckeana* toward AChE, exerted significantly (*p* < 0.05) higher anti‐AChE and anti‐BChE activity than their respective methanolic extracts, with the highest effect recorded, respectively, from the oil of *A. digitata* (2.44 and 2.10 mg GALAE/g, respectively) and *G. tenax* (2.18 and 2.03 GALAE/g, respectively). The three oils did not inhibit the Tyr enzyme, and the highest anti‐Tyr activity was exerted by the methanolic extract of *G. tenax* (10.88 mg KAE/g). The methanolic extract and oil of *T. garckeana* (0.71 and 0.63 mmol ACAE/g, respectively) displayed the best α‐amylase inhibition property, followed by those of *A. digitata* (0.40 and 0.43 mmol ACAE/g, respectively). The methanolic extract of *A. digitata* (1.46 mmol ACAE/g) revealed the highest α‐glucosidase inhibition, while the two extracts of *G. tenax* were not active. Previous enzyme inhibition studies were only performed for *A. digitata* and *T. garckeana* fruit pulp. Water, ethanol, and 70% ethanol extracts of the fruit pulp of *A. digitata* were recorded to exert significant anti‐AChE, anti‐Tyr, and α‐glucosidase inhibition activity, with values higher than those obtained for the seed in the present study (Hussain et al. 2019). Recently, six different *A. digitata* fruit pulp powders were evaluated using thin‐layer chromatography (HPTLC) hyphenated with an enzyme inhibition assay, and results showed that they exhibited significant anti‐AChE, anti‐BChE, anti‐Tyr, and α‐glucosidase inhibition activity, and these properties were assigned to palmitic, stearic, oleic, linoleic, and linolenic acids (Azadniya et al. 2023). Furthermore, the cholinesterase inhibition property by the oil could be attributed to linoleic acid (Akay et al. 2023). The hypoglycemic effect of *T. garckeana* fruit pulp was demonstrated from an in vitro and in vivo study (Lawal et al. 2022). The methanolic extract of the fruit pulp showed α‐amylase and α‐glucosidase inhibition activity in a dose‐dependent manner. Furthermore, it was reported that high amylase inhibition could be associated with intestinal discomfort, and thus superior glucosidase inhibition to amylase inhibition would be preferred (Cho et al. 2011). An in vivo study on streptozotocin‐induced diabetic rats showed that the methanolic extract of fruit pulp increased significantly the pancreatic insulin level. Thus, results of the current study showed that the seed of *T. garckeana* could also be a promising source of antidiabetic agents.

**TABLE 5:** Enzyme inhibition activity of oil and cake methanolic extracts of seeds.

Plant species	Extract	AChE (mg GALAE/g extract	BChE (mg GALAE/g extract)	Tyrosinase (mg KAE/g extract)	Amylase (mmol ACAE/g extract)	Glucosidase (mmol ACAE/g extract)
*Adansonia digitata*	Oil	2.44 ± 0.06^a^	2.10 ± 0.15^a^	NA	0.43 ± 0.00^c^	NA
	MeOH	1.83 ± 0.17^b^	1.45 ± 0.40^bc^	3.25 ± 0.84^b^	0.40 ± 0.01^c^	1.46 ± 0.00^a^
*Grewia tenax*	Oil	2.18 ± 0.05^ab^	2.03 ± 0.13^ab^	NA	0.18 ± 0.00^e^	NA
	MeOH	1.96 ± 0.18^b^	1.37 ± 0.27^cd^	10.88 ± 2.52^a^	0.28 ± 0.02^d^	NA
*Thespesia garckeana*	Oil	1.22 ± 0.15^c^	1.33 ± 0.22^cd^	NA	0.63 ± 0.00^b^	1.34 ± 0.02^b^
	MeOH	1.41 ± 0.23^c^	0.79 ± 0.06^d^	3.13 ± 0.84^b^	0.71 ± 0.03^a^	1.37 ± 0.03^b^

*Note:* Different letters in the same column indicate significant differences in the extracts (*p* < 0.05).

Abbreviations: ACAE, acarbose equivalent; GALAE, galantamine equivalent; KAE, Kojic acid equivalent; na, not active.

* Values are reported as mean ± SD of three parallel measurements.

## 4 Conclusion

The present study unveiled the nutraceutical potential of oilseeds of *Adansonia digita*, *Thespesia garckeana*, and *Grewia tenax*. The study also valorized the potential of their seedcakes that are usually considered waste products. Results indicated that these seeds could be promising candidates for the search for natural effective substances with antioxidant and enzyme inhibitory properties. *T. garckeana* and *G. tenax* were suggested as a promising sources of antioxidant substances. Besides, *G. tenax* could be a natural source for tyrosinase inhibitory molecules, while *A. digitata* could be a promising candidate for the isolation of cholinesterase and α‐glucosidase inhibitory molecules. Indeed, the three oils were predominated by essential fatty acids that cannot be synthesized by the human body. Thus, these three poorly explored species could be good sources of substances with various nutraceutical and pharmaceutical applications. To ensure their safety, toxicological studies are warranted. Also, the identification of active compounds and their mechanism of action are recommended.

## Author Contributions

**Munna Tahir Abdel Rahman:** methodology, investigation, data curation. **Sakina Yagi:** writing – original draft, writing – review and editing, investigation, data curation, conceptualization. **Gökhan Zengin:** writing – review and editing, writing – original draft, investigation, data curation, conceptualization. **Ozan Emre Eyupoglu:** methodology, investigation, data curation, conceptualization. **Rosella Spina:** writing – review and editing, data curation. **Jérémy Grosjean:** methodology, data curation. **Ashraf M. A. Abdalla:** validation, supervision, methodology. **Dominique Laurain‐Mattar:** writing – review and editing, data curation, conceptualization.

## Conflicts of Interest

The authors declare no conflicts of interest.

## Supporting information


Data S1.

## Data Availability

Data will be made available upon reasonable request.
