# Supplementary material for: Nutraceutical Potential of Oilseeds and By‐Products (Cakes) of Three Underutilized Malvaceae Trees Grown in Sudan
Source: Food Sci Nutr. 2025 Mar 5;13(3):e70080. doi: 10.1002/fsn3.70080 (PMC11882478; doi:10.1002/fsn3.70080)
Supplement: Supplementary file 1 — Data S1. [file FSN3-13-e70080-s001.docx]

Supplementary materials

*1. GC/MS analysis*

The chemical profile of oil seeds was determined by gas chromatography (GC) (Thermo Scientific Trace 1310) coupled with-mass spectrometry system (MS) (Thermo Scientific TSQ 9000 GC-MSMS) according to Hemmati et al. (2020). Analyses were performed using the equipment operating in the EI mode at 70 eV. An SLB5 column DB-5 ms (60 m, 0.25 mm film thickness) was employed with a 36 min temperature program of 60–320 at 10 °C/min followed by a 10 min hold at 320 °C. The injector temperature was 250 °C, the flow rate of the carrier gas (helium) was 1 mL/min, and the split ratio was 1:50. The interval of the scan m/z was between 35 and 900. The identity of different compounds was achieved by comparing the measured data with the NIST08.LIB database.

*2. Assays for total phenolic and flavonoid contents*

The total phenolic content was determined by employing the method given in the literature with some modification (Slinkard and Singleton, 1977). Sample solution (0.25 mL) was mixed with diluted Folin–Ciocalteu reagent (1 mL, 1:9, v/v) and shaken vigorously. After 3 min, Na2CO3 solution (0.75 mL, 1%) was added and the sample absorbance was read at 760 nm after a 2 h incubation at room temperature. The total phenolic contents were expressed as mg gallic acid equivalents (GAE)/g using the following equation based on the calibration curve: Y=0.0008x +0.0397 where x = concentration of gallic acid (100–900 mg/g) as standard.

The total flavonoid content was determined using the AlCl_3_ method (Slinkard and Singleton, 1977). Briefly, sample solution (1 mL) was mixed with the same volume of aluminum trichloride (2%) in methanol. Similarly, a blank was prepared by adding sample solution (1 mL) to methanol (1 mL) without AlCl3. The sample and blank absorbances were read at 415 nm after a 10 min incubation at room temperature. The absorbance of the blank was subtracted from that of the sample. Total flavonoids content was expressed as mg rutin equivalents (RE)/g using the following equation based on the calibration curve: Y= 0.0007x+0.0537, where x = concentration of rutin (100-900 mg/g) as standard.

*3. Determination of antioxidant effects*

Antioxidant (DPPH and ABTS radical scavenging, reducing power (CUPRAC and FRAP), phosphomolybdenum and metal chelating (ferrozine method)) activity was determined using the methods previously described by Grochowski et al. 2017. For the DPPH (1,1-diphenyl-2-picrylhydrazyl) radical scavenging assay: Sample solution was added to 4 mL of a 0.004% methanol solution of DPPH. The sample absorbance was read at 517 nm after a 30 min incubation at room temperature in the dark. DPPH radical scavenging activity was expressed as milligrams of trolox equivalents (mg TE/g extract). For ABTS (2,2′-azino-bis(3-ethylbenzothiazoline) 6-sulfonic acid) radical scavenging assay: Briefly, ABTS+ was produced directly by reacting 7 mM ABTS solution with 2.45 mM potassium persulfate and allowing the mixture to stand for 12–16 in the dark at room temperature. Prior to beginning the assay, ABTS solution was diluted with methanol to an absorbance of 0.700 ± 0.02 at 734 nm. Sample solution was added to ABTS solution (2 mL) and mixed. The sample absorbance was read at 734 nm after a 30 min incubation at room temperature. The ABTS radical scavenging activity was expressed as milligrams of trolox equivalents (mg TE/g extract). For CUPRAC (cupric ion reducing activity) activity assay: Sample solution was added to premixed reaction mixture containing CuCl2 (1 mL, 10 mM), neocuproine (1 mL, 7.5 mM) and NH4Ac buffer (1 mL, 1 M, pH 7.0). Similarly, a blank was prepared by adding sample solution (0.5 mL) to premixed reaction mixture (3 mL) without CuCl2 . Then, the sample and blank absorbances were read at 450 nm after a 30 min incubation at room temperature. The absorbance of the blank was subtracted from that of the sample. CUPRAC activity was expressed as milligrams of trolox equivalents (mg TE/g extract). For FRAP (ferric reducing antioxidant power) activity assay: Sample solution was added to premixed FRAP reagent (2 mL) containing acetate buffer (0.3 M, pH 3.6), 2,4,6- tris(2-pyridyl)-S-triazine (TPTZ) (10 mM) in 40 mM HCl and ferric chloride (20 mM) in a ratio of 10:1:1 (v/v/v). Then, the sample absorbance was read at 593 nm after a 30 min incubation at room temperature. FRAP activity was expressed as milligrams of trolox equivalents (mg TE/g extract). For phosphomolybdenum method: Sample solution was combined with 3 mL of reagent solution (0.6 M sulfuric acid, 28 mM sodium phosphate and 4 mM ammonium molybdate). The sample absorbance was read at 695 nm after a 90 min incubation at 95 °C. The total antioxidant capacity was expressed as millimoles of trolox equivalents (mmol TE/g extract). For metal chelating activity assay: Briefly, sample solution was added to FeCl2 solution (0.05 mL, 2 mM). The reaction was initiated by the addition of 5 mM ferrozine (0.2 mL). Similarly, a blank was prepared by adding sample solution (2 mL) to FeCl2 solution (0.05 mL, 2 mM) and water (0.2 mL) without ferrozine. Then, the sample and blank absorbances were read at 562 nm after 10 min incubation at room temperature. The absorbance of the blank was sub-tracted from that of the sample. The metal chelating activity was expressed as milligrams of EDTA (disodium edetate) equivalents (mg EDTAE/g extract). All spectrophotometric measurements were performed by using one spectrophotometer (UV-1800 , Shimadzu Corporation, Kyoto, Japan).

*4.On-Line HPLC methodologies*

*4.1 HPLC analysis*

LC-HPLC Agilent-1100 modular compact system (USA) with autosampler unit, UV-DAD, thermostable column cabinet with three trays utilized for the gradient analysis included combined a secondary syringe pump post-flown with reagent programmable quaternary channel injection (Inovenso IPS 13-RS laboratory model, Turkey). The experimental determinations were conducted utilizing encapped Purospher star, C_18_ column with guard-column (5 μm, 4.6 × 250 mm) (Germany, Merck). The injection volume of all samples was 20 μL. Analysis time was 30 min. Data investigations (retention time, peak area and detection limits) were executed utilizing with Chem-station of Agilent Program. The mobile phase contained of quaternary solvents in a harmony: solvent A was methanol, solvent B was a mixture of formic acid/ acetonitrile /aqua (3.5 / 48.25 / 48.25, v/v/v), solvent C was a mixture of 1.5 % formic acid: aqua (v/v) and solvent D was HPLC-grade acetonitrile (Sinan et al., 2021).

*4.2 HPLC-FRAP methodology*

HPLC-FRAP, post-column antioxidant detection methodology was utilized with fresh FRAP reagent (Benzie and Strain, 1999). Manager pump mobile phase flow rate was set at 0.7 mL / min. Detection signals were respectively set at max. 280 nm for DAD phenolic peaks and max. 595 nm for their UV antioxidant peaks. The flow rate of the secondary double channel injection pump was set at 0.25 mL / min (optimum) parallel to manager pump flow. Column oven was set at room temperature, 23-25°C. The reaction coil, which made of polytetrafluoroethylene tubing (0.22 mm i.d.), was adjusted as 1.5 m length (optimum). After FRAP reagent had been drawn into the syringe, covered with aluminum foil to protect from light, it was put into the secondary quaternary channel injection pump. Forms (0.5 mg / mL) of *Sakina* were analyzed with HPLC-FRAP methodology. They were filtered through a 0.20-micron filter prior to HPLC injection. They were run with HPLC-FRAP system at least three parallel each other. For this study, validation parameters about LOD and LOQ were respectively determined according to the International Conference on Harmonization guidelines as 3 times and 10 times of the average standard deviation of noise. UV 595 nm positive FRAP Peak chromatograms were simultaneously obtained with DAD 280 nm peak chromatograms. Linearity of the methodology was tested in the range of 15-85 ppm for detected 7 phenolic acids (Rutin, Apigenin, Quercetin, Pelargonidin, Kaempferol, Epigallocatechine gallate, Ferulic acid). The LOD and LOQ data determined for 595 nm and 280 nm separately. All statistical Agilent-Chem-station detection limit data and concentrations were reported significantly with standard deviations (*p*<0.05).

*4.3 HPLC-DPPH methodology*

HPLC-DPPH, also allows negative post-column detection of combining with determination of antioxidant activity and separation, was applied as scavenging activity with fresh DPPH radicalic reagent (Koleva et al., 2000). In the study, main pump and secondary pump flow parameters, column temperature parameters and solvent gradient ratios, sample injection concentrations used in HPLC-FRAP methodology were utilized exactly in this methodology without changing. Forms (0.5 mg / mL) of *Sakina* were analysed with HPLC-DPPH methodology. For this study, validation parameters about LOD and LOQ were respectively determined according to the International Conference on Harmonization guidelines as 3 times and 10 times of the average standard deviation of noise. UV 517 nm negative DPPH Peak chromatograms were simultaneously obtained with DAD 280 nm peak chromatograms. Linearity of the methodology was tested in the range of 11-65 ppm for detected 7 phenolic acids (Rutin, Apigenin, Quercetin, Pelargonidin, Kaempferol, Epigallocatechine gallate, Ferulic acid). The LOD and LOQ data determined for 517 nm and 280 nm separately. All statistical Agilent-Chem-station detection limit data and concentrations were reported significantly with standard deviations (*p*<0.05).

*4.4 HPLC-ABTS methodology*

HPLC-DPPH, also allows negative post-column detection of combining with determination of antioxidant activity and separation, was applied as scavenging activity with fresh ABTS radicalic reagent by Re et al. (1999). In the study, main pump and secondary pump flow parameters, column temperature parameters and solvent gradient ratios, sample injection concentrations used in HPLC-FRAP methodology were utilized exactly in this methodology without changing. Forms (0.5 mg / mL) of *Sakina* were analysed with HPLC-ABTS methodology. For this study, validation parameters about LOD and LOQ were respectively determined according to the International Conference on Harmonization guidelines as 3 times and 10 times of the average standard deviation of noise. UV 734 nm negative ABTS Peak chromatograms were simultaneously obtained with DAD 280 nm peak chromatograms. Linearity of the methodology was tested in the range of 22-77 ppm for detected 7 phenolic acids (Rutin, Apigenin, Quercetin, Pelargonidin, Kaempferol, Epigallocatechine gallate, Ferulic acid). The LOD and LOQ data determined for 734 nm and 280 nm separately. All statistical Agilent-Chem-station detection limit data and concentrations were reported significantly with standard deviations (*p*<0.05).

*4.5 HPLC-CUPRAC methodology*

HPLC-CUPRAC also allows negative post-column detection of combining with determination of antioxidant activity and separation, was applied with based on the reduction of Cu(II)-Neocuproine reagent in a redox reaction by Apak et al. (2004). In the study, main pump and secondary pump flow parameters, column temperature parameters and solvent gradient ratios, sample injection concentrations used in HPLC-FRAP methodology were utilized exactly in this methodology without changing. Forms (0.5 mg / mL) of *Sakina* were analysed with HPLC-CUPRAC methodology. For this study, validation parameters about LOD and LOQ were respectively determined according to the International Conference on Harmonization guidelines as 3 times and 10 times of the average standard deviation of noise. UV 450 nm negative CUPRAC Peak chromatograms were simultaneously obtained with DAD 280 nm peak chromatograms. Linearity of the methodology was tested in the range of 27-49 ppm for detected 7 phenolic acids (Rutin, Apigenin, Quercetin, Pelargonidin, Kaempferol, Epigallocatechine gallate, Ferulic acid). The LOD and LOQ data determined for 450 nm and 280 nm separately. All statistical Agilent-Chem-station detection limit data and concentrations were reported significantly with standard deviations (*p*<0.05).

*5. Enzyme inhibitory assays*

Enzyme inhibitory activities (cholinesterase (Elmann’s method), tyrosinase (dopachrome method), α-amylase (iodine/potassium iodide method), α -glucosidase (chromogenic PNPG method) and pancreatic lipase (p-nitrophenyl butyrate (p-NPB) method) were determined using the methods previously described by Uysal et al. (2017). The concentration range of samples were 0.5-5 mg/ml. For Cholinesterase (ChE) inhibitory activity assay: Sample solution (was mixed with DTNB (5,5-dithio-bis(2-nitrobenzoic) acid, Sigma, St. Louis, MO, USA) (125 µL) and AChE (acetylcholines-terase (Electric ell acetylcholinesterase, Type-VI-S, EC 3.1.1.7,Sigma)), or BChE (butyrylcholinesterase (horse serum butyrylcholinesterase, EC 3.1.1.8, Sigma)) solution (25 μL) in Tris–HCl buffer (pH 8.0) in a 96-well microplate and incubated for 15 min at 25 °C. The reaction was then initiated with the addition of acetylthiocholine iodide (ATCI, Sigma) or butyrylthiocholine chloride (BTCl, Sigma) (25 μL). Similarly, a blank was prepared by adding sample solution to all reaction reagents without enzyme (AChE or BChE) solution. The sample and blank absorbances were read at 405 nm after 10 min incubation at 25 °C. The absorbance of the blank was subtracted from that of the sample and the cholinesterase inhibitory activity was expressed as galanthamine equivalents (mgGALAE/g extract). For Tyrosinase inhibitory activity assay: Sample solution was mixed with tyrosinase solution (40 μL, Sigma) and phosphate buffer (100 μL, pH 6.8) in a 96-well microplate and incubated for 15 min at 25 °C. The reaction was then initiated with the addition of L-DOPA (40 μL, Sigma). Similarly, a blank was prepared by adding sample solution to all reaction reagents without enzyme (tyrosinase) solution. The sample and blank absorbances were read at 492 nm after a 10 min incubation at 25 °C. The absorbance of the blank was subtracted from that of the sample and the tyrosinase inhibitory activity was expressed as kojic acid equivalents (mg KAE/g extract). For α-amylase inhibitory activity assay: Sample solution was mixed with αamylase solution (ex-porcine pancreas, EC 3.2.1.1, Sigma) (50 μL) in phosphate buffer (pH 6.9 with 6 mM sodium chloride) in a 96-well microplate and incubated for 10 min at 37 °C. After pre-incubation, the reaction was initiated with the addition of starch solution (50 μL, 0.05%). Similarly, a blank was prepared by adding sample solution to all reaction reagents without enzyme (α-amylase) solution. The reaction mixture was incubated 10 min at 37 °C. The reaction was then stopped with the addition of HCl (25 μL, 1 M). This was followed by addition of the iodine-potassium iodide solution (100 μL). The sample and blank absorbances were read at 630 nm. The absorbance of the blank was subtracted from that of the sample and the α-amylase inhibitory activity was expressed as acarbose equivalents (mmol ACE/g extract). For α-glucosidase inhibitory activity assay: Sample solution was mixed with glutathione (50 µL), α-glucosidase solution (from Saccharomyces cerevisiae, EC 3.2.1.20, Sigma) (50 µL) in phosphate buffer (pH 6.8) and PNPG (4-N-trophenyl-α-D-glucopyranoside, Sigma) (50 µL) in a 96-well microplate and incubated for 15 min at 37 °C. Similarly, a blank was prepared by adding sample solution to all reaction reagents without enzyme (α-glucosidase) solution. The reaction was then stopped with the addition of sodium carbonate (50 µL, 0.2 M). The sample and blank absorbances were read at 400 nm. The absorbance of the blank was subtracted from that of the sample and the α-glucosidase inhibitory activity was expressed as acarbose equivalents (mmol ACE/g extract). All spectrophotometric measurements were performed by using one microplate reader (Multiskan-GO, Thermo Scientific).

References

Apak R, Güçlü K, Özyürek M, Karademir SE. Novel total antioxidant capacity index for dietary polyphenols and vitamins C and E, using their cupric ion reducing capability in the presence of neocuproine: CUPRAC method. J Agric Food Chem. 2004;52:7970–81.

Benzie, I. F., & Strain, J. J. (1999). Ferric reducing/antioxidant power assay: Direct measure of total antioxidant activity of biological fluids and modified version for simultaneous measurement of total antioxidant power and ascorbic acid concentration. Methods Enzymology, 299, 15–27.

Daniel M. Grochowski, Sengul Uysal, Abdurrahman Aktumsek, Sebastian Granica, Gokhan Zengin, Ramazan Ceylan, Marcello Locatelli, Michał Tomczyk, In vitro enzyme inhibitory properties, antioxidant activities, and phytochemical profile of Potentilla thuringiaca, Phytochemistry Letters, 20, 2017,365-372.

Hemmati, N., Azizi, M., Spina, R., Dupire, F., Arouei, H., Saeedi, M., Laurain-Mattar, D., 2020. Accumulation of ajmalicine and vinblastine in cell cultures is enhanced by endophytic fungi of Catharanthus roseus cv. Icy Pink. Ind. Crops Prod. 158, 112776.

Koleva, I. I., Niederländer, H. A., & van Been, T. A. (2000). An on-line HPLC method for detection of radical scavenging compounds in complex mixtures. Analytical Chemistry, 72(10), 2323–2328.

Re R, Pellegrini N, Proteggente A, Pannala A, Yang M, RiceEvans C. Antioxidant activity applying an improved ABTS radical cation decolorization assay. Free Radical Biol Med. 1999;26:1231–37.

Sinan, K. I., Mahomoodally, M. F., Eyupoglu, O. E., Etienne, O. K., Sadeer, N. B., Ak, G., Behl, T., & Zengin, G. (2021). HPLC-FRAP methodology and biological activities of different stem bark extracts of Cajanus cajan (L.) Millsp. Journal of Pharmaceutical and Biomed. Analysis, 192, 113678.

Slinkard, K.; Singleton, V. L. Total phenol analysis: automation and comparison with manual methods. Am. J. Enol. Vitic. 1977, 28 (1), 49−55.

Uysal, S., Zengin, G., Locatelli, M., Bahadori, M. B., Mocan, A., Bellagamba, G., & Aktumsek, A. (2017). Cytotoxic and enzyme inhibitory potential of two Potentilla species (P. speciosa L. and P. reptans Willd.) and their chemical composition. Frontiers in Pharmacology, 8, 290.

**Table S1.** Detection and Amount Limits of The Peaks Defined at 280 nm, 595 nm, 517 nm, 734 nm, 450 nm for Form of *Adansonia digitata*

| **Peak Numbers** | **Component Name** | **Retention Time (RT) (min.)** | **Concentrations (for 280 nm)**  **(ppm)** | | **Concentrations (for 595 nm)**  **(ppm)** | | **Concentrations (for 517 nm)**  **(ppm)** | | **Concentrations (for 734 nm)**  **(ppm)** | **Concentrations (for 450 nm)**  **(ppm)** | **Limit of Detection (LOD, ppm, 280 nm)** | **Limit of Quantitation (LOQ, ppm, 280 nm)** | **Limit of Detection (LOD, ppm, 595 nm)** | **Limit of Quantitation (LOQ, ppm, 595 nm)** | **Limit of Detection (LOD, ppm, 517 nm)** | **Limit of Quantitation (LOQ, ppm, 517 nm)** | **Limit of Detection (LOD, ppm, 734 nm)** | **Limit of Detection (LOQ, ppm, 734 nm)** | **Limit of Detection (LOD, ppm, 450 nm)** | **Limit of Detection (LOQ, ppm, 450 nm)** |
| --- | --- | --- | --- | --- | --- | --- | --- | --- | --- | --- | --- | --- | --- | --- | --- | --- | --- | --- | --- | --- |
| 1 | Rutin | 5.2 | 15±0.03 | | | 21.3±0.04 | | 19.5±0.02 | 20.5±0.03 | 23.2±0.04 | 22±0.02 | 66±0.04 | 33.1±0.03 | 109.3±0.04 | 17.1±0.01 | 51.3±0.03 | 18.4±0.03 | 55.2±0.04 | 19.1±0.02 | 57.3±0.04 |
| 2 | Apigenin | 10.4 | 57.1±0.04 | 62.1±0.04 | | | | 7.5±0.02 | 8.1±0.03 | 9±0.04 | 38.4±0.03 | 115.2±0.04 | 49.1±0.03 | 147.3±0.03 | 9.4±0.02 | 28.2±0.04 | 10.6±0.03 | 31.8±0.04 | 11.5±0.04 | 34.5±0.04 |
| 3 | Quercetin | 12.3 | 38.2±0.04 | 41.7±0.04 | | | | 9.5±0.02 | 11.2±0.03 | 14.2±0.04 | 46.4±0.03 | 139.2±0.04 | 57.1±0.03 | 171.3±0.04 | 10.5±0.03 | 31.5±0.04 | 11.5±0.03 | 34.5±0.04 | 13.5±0.02 | 40.5±0.04 |
| 4 | Pelargonidin | 16.5 | 82.5±0.03 | 85±0.04 | | | | 4.2±0.02 | 6.9±0.03 | 9±0.04 | 54.5±0.02 | 163.5±0.04 | 65.1±0.03 | 195.3±0.03 | 7.1±0.02 | 21.3±0.03 | 8.5±0.03 | 25.5±0.04 | 9.8±0.03 | 29.4±0.04 |
| 5 | Kaempferol | 18.3 | 40.1±0.04 | 43.1±0.04 | | | | 12.8±0.02 | 13.1±0.03 | 17±0.04 | 33.4±0.03 | 100.2±0.04 | 44.1±0.03 | 132.3±0.03 | 16.4±0.02 | 49.2±0.04 | 18.6±0.03 | 55.8±0.04 | 19.5±0.04 | 58.5±0.04 |
| 6 | Epigallocatechine gallate | 22.2 | 28.2±0.04 | 29.8±0.04 | | | | 14.5±0.02 | 16.2±0.03 | 18.2±0.04 | 21.4±0.03 | 64.2±0.04 | 32.3±0.03 | 96.9±0.04 | 16.8±0.03 | 50.4±0.04 | 19.4±0.03 | 57.2±0.04 | 21.5±0.02 | 64.5±0.04 |
| 7 | Ferulic acid | 24.5 | 67.5±0.04 | 78.7±0.04 | | | | 5.1±0.02 | 7.0±0.03 | 8.2±0.04 | 40.4±0.03 | 131.2±0.04 | 41.2±0.03 | 123.6±0.04 | 8.8±0.03 | 26.4±0.04 | 9.4±0.03 | 28.2±0.04 | 11.5±0.02 | 34.5±0.04 |

^±^SD: Average Standard Deviation, 95 % confidence interval, critical ratio: *p*<0.05, ppm: parts per million

**Table S2.** Detection and Amount Limits of The Peaks Defined at 280 nm, 595 nm, 517 nm, 734 nm, 450 nm for Form of *Thespesia garckeana* F.Hoffm. (syn*. Azanza garckeana*)*.*

| **Peak Numbers** | **Component Name** | **Retention Time (RT) (min.)** | **Concentrations (for 280 nm)**  **(ppm)** | | **Concentrations (for 595 nm)**  **(ppm)** | | **Concentrations (for 517 nm)**  **(ppm)** | | **Concentrations (for 734 nm)**  **(ppm)** | **Concentrations (for 450 nm)**  **(ppm)** | **Limit of Detection (LOD, ppm, 280 nm)** | **Limit of Quantitation (LOQ, ppm, 280 nm)** | **Limit of Detection (LOD, ppm, 595 nm)** | **Limit of Quantitation (LOQ, ppm, 595 nm)** | **Limit of Detection (LOD, ppm, 517 nm)** | **Limit of Quantitation (LOQ, ppm, 517 nm)** | **Limit of Detection (LOD, ppm, 734 nm)** | **Limit of Detection (LOQ, ppm, 734 nm)** | **Limit of Detection (LOD, ppm, 450 nm)** | **Limit of Detection (LOQ, ppm, 450 nm)** |
| --- | --- | --- | --- | --- | --- | --- | --- | --- | --- | --- | --- | --- | --- | --- | --- | --- | --- | --- | --- | --- |
| 1 | Rutin | 5.2 | 14±0.03 | | | 20.3±0.04 | | 18.5±0.02 | 19.5±0.03 | 21.2±0.04 | 21±0.02 | 63±0.04 | 32.1±0.03 | 96.3±0.04 | 16.1±0.01 | 48.3±0.03 | 17.4±0.03 | 52.2±0.04 | 18.1±0.02 | 54.3±0.04 |
| 2 | Apigenin | 10.4 | 55.1±0.04 | 60.1±0.04 | | | | 6.5±0.02 | 7.1±0.03 | 8±0.04 | 37.4±0.03 | 112.2±0.04 | 48.1±0.03 | 144.3±0.03 | 8.4±0.02 | 25.2±0.04 | 9.6±0.03 | 28.8±0.04 | 10.5±0.04 | 31.5±0.04 |
| 3 | Quercetin | 12.3 | 36.2±0.04 | 40.7±0.04 | | | | 8.5±0.02 | 10.2±0.03 | 13.2±0.04 | 45.4±0.03 | 136.2±0.04 | 56.1±0.03 | 168.3±0.04 | 9.5±0.03 | 28.5±0.04 | 10.5±0.03 | 31.5±0.04 | 12.5±0.02 | 37.5±0.04 |
| 4 | Pelargonidin | 16.5 | 80.5±0.03 | 83±0.04 | | | | 3.2±0.02 | 5.9±0.03 | 8±0.04 | 53.5±0.02 | 160.5±0.04 | 64.1±0.03 | 192.3±0.03 | 6.1±0.02 | 18.3±0.03 | 7.5±0.03 | 22.5±0.04 | 8.8±0.03 | 25.4±0.04 |
| 5 | Kaempferol | 18.3 | 39.1±0.04 | 41.1±0.04 | | | | 11.8±0.02 | 12.1±0.03 | 16±0.04 | 30.4±0.03 | 91.2±0.04 | 43.1±0.03 | 129.3±0.03 | 15.4±0.02 | 46.2±0.04 | 17.6±0.03 | 52.8±0.04 | 18.5±0.04 | 55.5±0.04 |
| 6 | Epigallocatechine gallate | 22.2 | 25.2±0.04 | 27.8±0.04 | | | | 13.5±0.02 | 15.2±0.03 | 17.2±0.04 | 20.4±0.03 | 61.2±0.04 | 31.3±0.03 | 93.9±0.04 | 15.8±0.03 | 47.4±0.04 | 18.4±0.03 | 55.2±0.04 | 20.5±0.02 | 61.5±0.04 |
| 7 | Ferulic acid | 24.5 | 65.5±0.04 | 74.7±0.04 | | | | 4.1±0.02 | 6.0±0.03 | 7.2±0.04 | 39.4±0.03 | 117.2±0.04 | 40.2±0.03 | 120.6±0.04 | 7.8±0.03 | 23.4±0.04 | 8.4±0.03 | 25.2±0.04 | 10.5±0.02 | 31.5±0.04 |

^±^SD: Average Standard Deviation, 95 % confidence interval, critical ratio: *p*<0.05, ppm: parts per million

**Table S3.** Detection and Amount Limits of The Peaks Defined at 280 nm, 595 nm, 517 nm, 734 nm, 450 nm for Form of *Grewia tenax*.

| **Peak Numbers** | **Component Name** | **Retention Time (RT) (min.)** | **Concentrations (for 280 nm)**  **(ppm)** | | **Concentrations (for 595 nm)**  **(ppm)** | | **Concentrations (for 517 nm)**  **(ppm)** | | **Concentrations (for 734 nm)**  **(ppm)** | **Concentrations (for 450 nm)**  **(ppm)** | **Limit of Detection (LOD, ppm, 280 nm)** | **Limit of Quantitation (LOQ, ppm, 280 nm)** | **Limit of Detection (LOD, ppm, 595 nm)** | **Limit of Quantitation (LOQ, ppm, 595 nm)** | **Limit of Detection (LOD, ppm, 517 nm)** | **Limit of Quantitation (LOQ, ppm, 517 nm)** | **Limit of Detection (LOD, ppm, 734 nm)** | **Limit of Detection (LOQ, ppm, 734 nm)** | **Limit of Detection (LOD, ppm, 450 nm)** | **Limit of Detection (LOQ, ppm, 450 nm)** |
| --- | --- | --- | --- | --- | --- | --- | --- | --- | --- | --- | --- | --- | --- | --- | --- | --- | --- | --- | --- | --- |
| 1 | Rutin | 5.2 | 11±0.03 | | | 17.3±0.04 | | 15.5±0.02 | 16.5±0.03 | 18.2±0.04 | 18±0.02 | 54±0.04 | 29.1±0.03 | 87.3±0.04 | 13.1±0.01 | 39.3±0.03 | 14.4±0.03 | 43.2±0.04 | 15.1±0.02 | 45.3±0.04 |
| 2 | Apigenin | 10.4 | 52.1±0.04 | 57.1±0.04 | | | | 3.5±0.02 | 4.1±0.03 | 5±0.04 | 34.4±0.03 | 103.2±0.04 | 45.1±0.03 | 135.3±0.03 | 5.4±0.02 | 16.2±0.04 | 6.6±0.03 | 19.8±0.04 | 7.5±0.04 | 22.5±0.04 |
| 3 | Quercetin | 12.3 | 33.2±0.04 | 37.7±0.04 | | | | 5.5±0.02 | 7.2±0.03 | 10.2±0.04 | 42.4±0.03 | 127.2±0.04 | 53.1±0.03 | 159.3±0.04 | 6.5±0.03 | 19.5±0.04 | 7.5±0.03 | 22.5±0.04 | 9.5±0.02 | 28.5±0.04 |
| 4 | Pelargonidin | 16.5 | 77.5±0.03 | 80±0.04 | | | | 0.25±0.02 | 2.9±0.03 | 5±0.04 | 50.5±0.02 | 151.5±0.04 | 61.1±0.03 | 183.3±0.03 | 3.1±0.02 | 9.3±0.03 | 4.5±0.03 | 13.5±0.04 | 5.8±0.03 | 16.4±0.04 |
| 5 | Kaempferol | 18.3 | 36.1±0.04 | 38.1±0.04 | | | | 8.8±0.02 | 9.1±0.03 | 13±0.04 | 27.4±0.03 | 82.2±0.04 | 40.1±0.03 | 120.3±0.03 | 12.4±0.02 | 37.2±0.04 | 14.6±0.03 | 43.8±0.04 | 15.5±0.04 | 46.5±0.04 |
| 6 | Epigallocatechine gallate | 22.2 | 22.2±0.04 | 24.8±0.04 | | | | 10.5±0.02 | 12.2±0.03 | 14.2±0.04 | 17.4±0.03 | 52.2±0.04 | 28.3±0.03 | 84.9±0.04 | 12.8±0.03 | 38.4±0.04 | 15.4±0.03 | 46.2±0.04 | 17.5±0.02 | 52.5±0.04 |
| 7 | Ferulic acid | 24.5 | 62.5±0.04 | 71.7±0.04 | | | | 1.1±0.02 | 3.0±0.03 | 4.2±0.04 | 36.4±0.03 | 109.2±0.04 | 37.2±0.03 | 111.6±0.04 | 4.8±0.03 | 14.4±0.04 | 5.4±0.03 | 16.2±0.04 | 7.5±0.02 | 22.5±0.04 |

^±^SD: Average Standard Deviation, 95 % confidence interval, critical ratio: *p*<0.05, ppm: parts per million

**Table S4.** Full validation analysis results for 4 different on-line HPLC-Antioxidant methods of detected phenolic acids as external standards

| **Methods** | **Compounds** | **LOD (ppm±SD)** | **LOQ (ppm±SD)** | **Specificity (%)** | **Matrix Effect (%)** | **Interday Precision (%)** | **Intraday Precision (%)** | **Recovery (%)** | **Calibration Curve Equations** | **Repeatability (%)** | **R²** |
| --- | --- | --- | --- | --- | --- | --- | --- | --- | --- | --- | --- |
| **On-line HPLC-FRAP** | Rutin | 26.2±0.04 | 78.6±0.08 | 0.7 | 75.2 | 5.5 | 1.8 | 98.5 | 𝑦 =47.39𝑥−12500 | 99.8 | 0.9992 |
|  | Apigenin | 25.9±0.11 | 77.7±0.15 | 1.7 | 77.8 | 6.6 | 2.4 | 102.1 | 𝑦 =37.69𝑥−12840 | 99.6 | 0.9991 |
|  | Quercetin | 31.3±0.35 | 93.9±0.25 | 2.1 | 76.8 | 8.1 | 3.5 | 100.1 | 𝑦 =83.84𝑥−17640 | 99.5 | 0.9990 |
|  | Pelargonidin | 20.4±0.12 | 61.2±0.19 | 3.4 | 78.4 | 9.2 | 4.2 | 99.4 | 𝑦 =65.85𝑥−19500 | 99.4 | 0.9989 |
|  | Kaempferol | 6.7±0.20 | 20.1±0.17 | 1.6 | 79.4 | 7.8 | 4.1 | 98.7 | 𝑦 =57.79𝑥−21500 | 99.7 | 0.9988 |
|  | Epigallocatechine gallate | 5.8±0.07 | 17.4±0.09 | 3.5 | 76.7 | 8.4 | 3.9 | 103.2 | 𝑦 =71.59𝑥−11500 | 99.1 | 0.9987 |
|  | Ferulic acid | 15.5±0.28 | 46.5±0.30 | 2.8 | 74.9 | 7.9 | 2.8 | 104.1 | 𝑦 =39.89𝑥−18500 | 99.0 | 0.9986 |
| **On-line HPLC-DPPH** | **Compounds** | **LOD (ppm±SD)** | **LOQ (ppm±SD)** | **Specificity (%)** | **Matrix Effect (%)** | **Interday Precision (%)** | **Intraday Precision (%)** | **Recovery (%)** | **Calibration Curve Equations** | **Repeatability (%)** | **R²** |
|  | Rutin | 11.2±0.04 | 33.6±0.04 | 1.5 | 82.4 | 6.5 | 1.7 | 97.5 | 𝑦=98.2𝑥−12750 | 98.8 | 0.9985 |
|  | Apigenin | 21.5±0.05 | 64.5±0.05 | 2.3 | 81.7 | 4.7 | 1.9 | 100.1 | 𝑦=104.2𝑥−13550 | 98.6 | 0.9984 |
|  | Quercetin | 44.7±0.15 | 134.1±0.25 | 3.6 | 83.4 | 4.3 | 2.3 | 99.1 | 𝑦=61.4𝑥−23750 | 98.5 | 0.9983 |
|  | Pelargonidin | 37.4±0.12 | 82.2±0.17 | 4.1 | 86.7 | 8.5 | 3.7 | 98.4 | 𝑦=75.6𝑥−32750 | 98.4 | 0.9982 |
|  | Kaempferol | 14.8±0.22 | 44.4±0.32 | 2.2 | 89.5 | 3.8 | 3.4 | 99.7 | 𝑦=218.2𝑥−49950 | 98.7 | 0.9981 |
|  | Epigallocatechine gallate | 3.8±0.14 | 11.4±0.14 | 0.9 | 82.4 | 8.7 | 3.1 | 101.2 | 𝑦=33.5𝑥−29850 | 98.1 | 0.9980 |
|  | Ferulic acid | 9.5±0.18 | 28.5±0.28 | 1.9 | 81.1 | 7.6 | 2.9 | 100.1 | 𝑦=42.7𝑥−52750 | 98.0 | 0.9979 |
| **On-line HPLC-CUPRAC** | **Compounds** | **LOD (ppm±SD)** | **LOQ (ppm±SD)** | **Specificity (%)** | **Matrix Effect (%)** | **Interday Precision (%)** | **Intraday Precision (%)** | **Recovery (%)** | **Calibration Curve Equations** | **Repeatability (%)** | **R²** |
|  | Rutin | 10.3±0.04 | 30.9±0.14 | 1.4 | 71.5 | 5.4 | 2.2 | 96.5 | 𝑦=22.7𝑥−42750 | 97.8 | 0.9978 |
|  | Apigenin | 31.4±0.17 | 94.2±0.16 | 1.8 | 75.4 | 7.2 | 3.1 | 100.1 | 𝑦=142.7𝑥−891750 | 97.6 | 0.9977 |
|  | Quercetin | 24.6±0.05 | 73.8±0.15 | 3.8 | 72.9 | 6.8 | 4.4 | 90.1 | 𝑦=62.9𝑥−92750 | 97.5 | 0.9976 |
|  | Pelargonidin | 17.1±0.19 | 51.3±0.27 | 2.7 | 76.8 | 4.5 | 4.3 | 95.4 | 𝑦=32.5𝑥−72850 | 97.4 | 0.9975 |
|  | Kaempferol | 4.9±0.02 | 14.7±0.06 | 4.2 | 77.5 | 8.9 | 2.8 | 94.7 | 𝑦=42.7𝑥−52750 | 97.7 | 0.9974 |
|  | Epigallocatechine gallate | 8.8±0.24 | 26.4±0.04 | 3.6 | 76.4 | 7.5 | 2.6 | 105.2 | 𝑦=49.7𝑥−62780 | 97.1 | 0.9973 |
|  | Ferulic acid | 7.5±0.08 | 22.5±0.16 | 2.4 | 72.8 | 8.3 | 1.9 | 103.1 | 𝑦=92.7𝑥−82950 | 97.0 | 0.9972 |
| **On-line HPLC-ABTS** | **Compounds** | **LOD (ppm±SD)** | **LOQ (ppm±SD)** | **Specificity (%)** | **Matrix Effect (%)** | **Interday Precision (%)** | **Intraday Precision (%)** | **Recovery (%)** | **Calibration Curve Equations** | **Repeatability (%)** | **R²** |
|  | Rutin | 21.3±0.24 | 63.9±0.34 | 1.3 | 83.4 | 7.4 | 2.5 | 95.5 | 𝑦=112.3𝑥−87450 | 99.8 | 0.9971 |
|  | Apigenin | 39.4±0.13 | 118.2±0.18 | 1.1 | 85.7 | 7.1 | 3.5 | 99.1 | 𝑦=96.6𝑥−22790 | 99.6 | 0.9970 |
|  | Quercetin | 34.7±0.05 | 104.1±0.15 | 2.7 | 88.2 | 6.9 | 4.6 | 97.1 | 𝑦=43.7𝑥−72770 | 99.5 | 0.9969 |
|  | Pelargonidin | 7.1±0.09 | 21.3±0.07 | 2.3 | 89.5 | 5.8 | 4.1 | 96.4 | 𝑦=57.4𝑥−72790 | 99.4 | 0.9968 |
|  | Kaempferol | 2.9±0.02 | 8.7±0.06 | 3.1 | 84.4 | 5.7 | 3.9 | 102.7 | 𝑦=63.7𝑥−92950 | 99.7 | 0.9967 |
|  | Epigallocatechine gallate | 5.3±0.04 | 15.9±0.16 | 2.5 | 86.5 | 4.6 | 3.7 | 93.2 | 𝑦=25.6𝑥−82780 | 99.1 | 0.9966 |
|  | Ferulic acid | 9.5±0.28 | 28.5±0.26 | 3.7 | 83.7 | 8.2 | 3.5 | 98.1 | 𝑦=22.7𝑥−32790 | 99.0 | 0.9965 |

Phenolic acids were validated through a comprehensive analysis encompassing several key parameters for four on-line HPLC-antioxidant methodology. Precision included both repeatability (intra-day precision) and intermediate precision (inter-day precision), evaluated by calculating the % relative standard deviation (RSD) of multiple measurements. Specificity was ensured by analyzing samples containing potential interferences and confirming no significant signal overlap with the analyte. Repeatability was assessed by the consistency of results under the same conditions over a short period, using RSD. Finally, % recovery was evaluated by comparing the amount of phenolic acids recovered from the sample matrix to the amount initially spiked, confirming the method’s robustness and suitability for accurate quantification of phenolic acids (González-González et. al., 2019) (Table S4).

González-González, R. M., Barragán-Mendoza, L., Peraza-Campos, A. L., Muñiz-Valencia, R., & Ceballos-Magaña, S. G. (2019). Validation of an HPLC-DAD method for the determination of plant phenolics. *Revista Brasileira de Farmacognosia*, 29(6), 689-693. https://doi.org/10.1016/j.bjp.2019.06.002
